# A cloud computing based 12-lead ECG telemedicine service

**DOI:** 10.1186/1472-6947-12-77

**Published:** 2012-07-28

**Authors:** Jui-chien Hsieh, Meng-Wei Hsu

**Affiliations:** 1 Department of Information Management, Yuan Ze Uiversity, 135 Yuan-Tung Road, Chungli 32003, Taiwan

**Keywords:** ECG, Telecardiology, Cloud computing, Pervasive computing

## Abstract

**Background:**

Due to the great variability of 12-lead ECG instruments and medical specialists’ interpretation skills, it remains a challenge to deliver rapid and accurate 12-lead ECG reports with senior cardiologists’ decision making support in emergency telecardiology.

**Methods:**

We create a new cloud and pervasive computing based 12-lead Electrocardiography (ECG) service to realize ubiquitous 12-lead ECG tele-diagnosis.

**Results:**

This developed service enables ECG to be transmitted and interpreted via mobile phones. That is, tele-consultation can take place while the patient is on the ambulance, between the onsite clinicians and the off-site senior cardiologists, or among hospitals. Most importantly, this developed service is convenient, efficient, and inexpensive.

**Conclusions:**

This cloud computing based ECG tele-consultation service expands the traditional 12-lead ECG applications onto the collaboration of clinicians at different locations or among hospitals. In short, this service can greatly improve medical service quality and efficiency, especially for patients in rural areas. This service has been evaluated and proved to be useful by cardiologists in Taiwan.

## Background

### Clinically-used 12-Lead ECG

The 12-lead Electrocardiography (ECG) is one of the most frequently applied diagnostic tools in clinical cardiac medicine. A 12-lead ECG instrument records electrical activities of the heart from the frontal and the horizontal views by attaching 10 electrodes on the surfaces of limbs and the chest and therefore generates 12 groups of signals, called 12-lead ECG. Traditionally, a 12-lead ECG instrument stores ECG waveform data with vendor-specific formats whose ECG data are compressed or encrypted with vendor-specific algorithms in the instrument and then generates a hard-copy of the 12-lead ECG report. Recently, ECG instruments are equipped with vendor-specific computerized ECG viewers, which can be used only on desktop computers. In Taiwan, most hospitals adopt paper-ECG, which is due to the expenses of vender-specific ECG instruments and ECG viewers, as well as the management problems of 12-lead ECG records stored in different systems provided by various 12-lead ECG manufactures.

### From paper-ECG to computerized ECG

In 2002, Open-ECG, an international academic organization, promoted the development of computerized 12-lead ECG by providing researchers with technical references of 12-lead ECG data formats, including International Organization for Standardization (ISO) approved Standard Communication Protocol–ECG (SCP-ECG), Food and Drug Administration (FDA) proposed Extensible Markup Language based ECG (XML-ECG), and National Electrical Manufactures Association (NEMA) recommended Digital Imaging and Communications in Medicine based ECG (DICOM-ECG) [1-4]. However, several ECG manufactures do not completely adopt the open protocol standards. Instead, they develop vendor-specific ECG data formats and ECG waveform encoding rules. Consequently, 12-lead ECG data formats are heterogeneous and vendor-dependent in clinical practice. With the help of OPEN-ECG, hospitals can develop 12-lead ECG e-diagnosis instead of paper-ECG by extracting the waveform data from several ECG instruments [5,6].

### The integration of heterogeneous 12-lead ECG

When a hospital purchases various ECG products from different manufactures, not only do clinicians encounter huge difficulty in retrieving ECG records, but information technicians (ITs) also have great difficulty in managing ECG information systems due to the heterogeneous ECG data formats and incompatible ECG information database provided by 12-lead ECG manufactures. To provide clinicians and ITs with easier ECG management by unifying heterogeneous ECG data formats, researchers converted clinically-used SCP-ECG and XML-ECG to DICOM-ECG that can be integrated in the Picture Archiving and Communication Systems (PACS) with medical images [7].

### 12-lead ECG telemedicine and pervasive health

The development of mobile computing-based medical applications is crucial, because it proves to enhance medical service quality [8,9]. European M-Health Alliance (EuMHA) is a non-profit organization established in Finland in 2010. The goals of EuMHA are to promote the current health IT and to enhance the health service quality with various m-health applications and products [10]. To date, several mobile based single-lead or three-lead ECG products have been successfully developed to monitor heart rhythms remotely [11,12]. A pioneer ECG telemedicine project, “Enhanced Personal, Intelligent, and Mobile system for Early Detection and Interpretation of Cardiac Syndromes (EPI-MEDICS)”, was created to change the traditional hospital-based cardiac care services to the personalized and non-hospital-based cardiac telecare services [13,14]. In this European project, a self-developed ECG device, Personal Electrocardiogram Monitor (PEM), has the following functions: (a) synthesizing 12-lead ECG from the measured 3-lead ECG, (b) storing serial ECGs in the standard SCP-ECG format, (c) storing personal health record (PHR) in the XML format, (d) containing artificial intelligence based ECG interpretation, and (e) transmitting ECGs with PHR to remote care providers via Global System for Mobile Communication (GSM). This device greatly helped enhance the quality of cardiac telecare services because remote cardiologists can therefore offer timely diagnosis and treatment order for patients with heart diseases. Additionally, the integration of serial SCP-ECGs allows the cardiologists to access patients’ past and current ECG records, as well as PHR, which greatly facilitate the process of performing diagnosis and treatment with more comprehensive references to patients’ medical records [15,16].

However, clinically used 12-lead ECG instruments are limited to the use in the hospital and consequently, they do not generate ECG reports outside the hospital. Due to the great variability of 12-lead ECG instruments and medical specialists’ interpretation skills, it remains a challenge to deliver rapid and accurate 12-lead ECG reports with senior cardiologists’ decision making support [17]. In fact, most 12-lead ECG telemedicine devices lack the ability to integrate with clinically-used 12-lead ECG instruments which generate reports and Hospital Information System (HIS) containing patients’ medication information and lab test reports. To enable 12-lead ECG transmission, a hospital has to purchase an additional ECG telemedicine station to receive ECG transmitted from ambulances, and a cardiologist has to be present at the station to interpret the transmitted ECG reports. Nevertheless, it is not always the case that a senior cardiologist stays in the hospital. When a patient needs immediate treatment and medical staff needs to consult a cardiologist who is physically away from the hospital, current 12-lead ECG telemedicine system cannot provide this cardiologist with patients’ historic ECG records, lab test results, or medication records. Consequently, medial services are greatly compromised.

**Figure 1:**
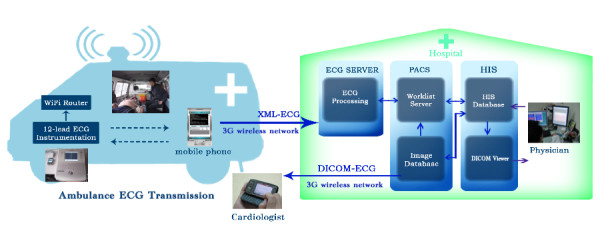
** The 12-lead ECG telemedicine applications in clinical practice.** As the clinically-used ECG instrument generating reports in the XML format is integrated into PACS, clinicians of the hospital can interpret ECG via PACS and HIS. Pre-hospital 12-lead ECG diagnosis can be made as a result of the delivery of ECG reports between the ECG instruments in the ambulance and in the hospital. In addition, off-site cardiologists can make ubiquitous tele-diagnosis through the ECG database connection via their cell phones.

To resolve the aforementioned problems, we developed a series studies in 2009 and 2010 as illustrated in Figure 1. In these studies, we improved the traditional 12-lead ECG telemedicine through the use of mobile computing [6,7,18]. In these studies, the off-site cardiologists can ubiquitously use their cell phones implemented with mobile database to connect with hospitals’ database and to access patients’ ECG e-reports and medication records stored in HIS, so that they can offer timely assistance to the on-site Emergency Department (ED) physicians to deliver the most appropriate intervention. The clinically-used 12-lead ECG instrument is also transformed into a portable device that can be installed in an ambulance. The emergency medical technician (EMT) in the ambulance can use a cell phone equipped with Wi-Fi and 3G wireless telecommunication modules to deliver ECG to the hospital and the cell phones of off-site senior cardiologists in real time. It should be noted that the transmitted ECG report from an ambulance is then integrated in PACS and HIS system, so that off-site cardiologist can rapidly perform pre-hospital diagnosis with sufficient references and reduce the “door-to-balloon” time, which means the time elapses starting from the patient’s arrival in hospital and ending when a catheter crosses the blocked coronary artery. The mobile computing based 12-lead ECG telemedicine as compared to the traditional ECG telemedicine has great advantages because it is mobile and easy to use. More importantly, this system ensures rapid transmission between devices outside and inside the hospital, and it enables off-site senior cardiologist to ubiquitously access 12-lead ECG reports and offer timely decision making support.

Based on recent investigation in 2011 [19,20], there are three major challenges in 12-lead ECG telemedicine, including (1) How can a patient’s 12-lead ECG measured in a moving ambulance or obtained at home be transmitted to the admitting hospital with rapid and effective pre-hospital diagnosis ? (2) How can the medical staff in rural areas use an open and public tele-cardiology system to consult experienced cardiologists in real time to improve treatment in emergency situations? (3) How to set up an open platform for researchers to perform large-scale 12-lead ECG clinical trials? In addition, 12-lead ECG interoperability is crucial to emergency telemedicine, especially if a patient with acute myocardial infarction is referred from one hospital to another hospital. When the cardiologist can interpret both current and past ECG from the previous hospital via shared medical system, repeated ECG examinations can be reduced and the patient’s treatment plan can be established appropriately and efficiently. However, 12-lead ECG interoperability is difficult to establish because of the heterogeneous HIS and ECG data formats [21].

The present challenges of 12-lead ECG telemedicine include not only to improve the services of a single clinic but also to strengthen the collaboration among clinics such as inter-hospital ECG interpretation. A promising method to develop inter-hospital 12-lead ECG telemedicine is to use cloud computing technology providing hospitals with a common platform with easily accessible computing resources and storage space on demand via internet and a low-cost pay-per-use model without long-term commitment.

### Cloud computing, pervasive computing, and telemedicine

Cloud computing refers to the software application delivery service via web access among different computers with heterogeneous O.S. [22]. The cloud providers, who own large datacenters composed of a large amount of computers with internet link, provide users with the environment of data storage and software development with abstract management of computing resources in the datacenters. The user-developed applications in the cloud can be registered as services to the public. An individual or an organization can subscribe the cloud services to use the applications via web access without any hardware or software installation on-premise computers. Many applications, such as web hosting, data archiving, large-scale simulations, and social networking, can benefit from its features, including easily accessible computing resources and storage space on demand, networking, and high performance computing. Recent studies indicated that cloud computing can improve healthcare services and benefit biomedical research. For instance, a cloud computing based Voice over IP (VoIP) service for diabetic patients’ self-care management is created in 2010 by Piette and his colleagues [23]. The patients subscribing the cloud service received VoIP calls with pre-recorded voice messages as self-care reminders. In this study, the participants obtained better glycemic control than the patients without subscribing the service. The advantages of this cloud computing based healthcare service are cost effective, and it can be extended globally with ease. Recent studies also indicated that cloud computing can facilitate the biomedical informatics research communities, which need large-scale shared data and computational tools. Fusaro and his colleagues (2011) used high performance computing of Amazon Web Service to facilitate genomic findings [24]. There was other research proposing concept framework to improve healthcare services through the collaboration of cloud computing and mobile computing. For example, Nkosi and his colleagues (2010) proposed cloud computing based framework to improve deficient mobile devices used for healthcare services, including computing capacity, memory, and power consumption [25]. In this study, the mobile devices were used to acquire physiological signals, which were processed with massive computing resources. To relieve heavy execution and avoid power loss of mobile devices, the acquired physiological signals were redirected to a cloud service performing signal processing. The mobile devices then automatically received the results if the cloud service finished the job of massive computing of signal processing. This technology can benefit patients living at home whose physiological signals need to be monitored continuously, as their cell phones can transmit their physiological signals to a cloud service, which is responsible for signal processing, and remote physicians can access the results via web with a desktop computer or a cell phone. An industrial project of cloud computing based tele-homecare service is setting up by IBM, several European academic, research organizations, and hospitals. In this project, the patient’s vital signals can be monitored at home, and patients’ medication records are stored in a centralized cloud database. The physician can use the cloud platform to diagnose patients at home, the patients can access their medical records via this cloud service, and pharmacy administers can monitor and control the use of drugs [26].

### The objectives of this study

This study aims to develop an effective model of 12-lead ECG telemedicine to overcome the challenges of 12-lead ECG telemedicine described in Sec. of 12-lead ECG telemedicine and pervasive health. The objectives of this study are as follows, (a) to facilitate inter-hospital 12-lead ECG tele-consultation, (b) to practice pre-hospital diagnosis, and (c) to enhance the interoperability of 12-lead ECG records among urban hospitals and rural clinics without the costly installation of additional 12-lead ECG telemedicine equipment.

## Methods

### 12-lead ECG infrastructure

In this study, we developed an ECG telemedicine service from clinically used ECG devices with heterogeneous data formats, such as HP compatible SCP-ECG, Mortara DICOM-ECG and Philips XML-ECG, which are commonly used in both urban and rural areas in Taiwan. Most modern 12-lead ECG devices in clinical practice can directly export ECG reports via wired or wireless based network, such as Philips ECG XML-ECG, or indirectly export ECGs via internet through the transformation from the traditional serial transmission, RS232 or USB, to Ethernet based network, such as HP compatible SCP-ECG device and Mortara DICOM-ECG device. In this study, the Ethernet based network with an average speed ranging from 15 Mbps to 80 Mbps via TCP/IP protocol was used to transmit and receive ECGs between the cloud service and ECG devices either at a metropolitan hospital or at a rural clinic. A 3G mobile telephone network allowing ECG delivery with the speed of 1.5 ~ 3.6 Mbps was used between the cloud service and the cardiologist’s mobile phone to realize ubiquitous 12-lead ECG tele-consultation. This cloud ECG service can directly receive various ECG files from ECG devices generating ECGs via TCP/IP based network and allow a clinician to browse ECG reports via Web, which operates independently from the hospital O.S.

### Cloud based 12-lead ECG telemedicine system

**Figure 2:**
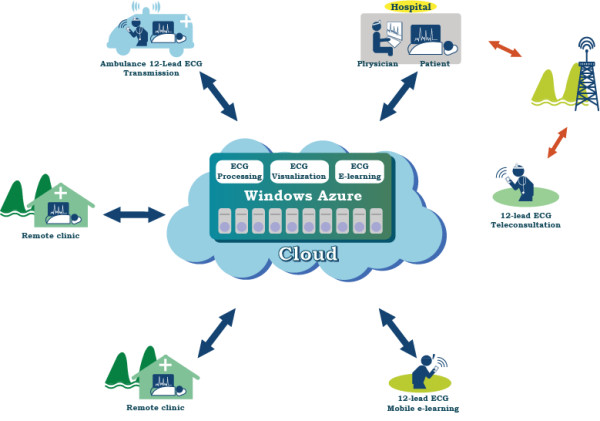
** A 12-lead ECG telemedicine service based on the cloud computing.** The heterogeneous 12-lead ECG files from urban hospitals, rural clinics, and ambulances can transmit and store 12-lead ECG files in the datacenter of Windows Azure with unified file structure via internet. The ECG interoperability among hospitals can be easily realized by sharing ECG records in the cloud. In addition, clinicians can use these 12-lead ECG records in the cloud for consultations or as e-learning materials of clinical ECG continuing education.

This cloud computing based 12-lead information system and telemedicine applications in clinical practice are illustrated in Figure 2. A 12-lead ECG telemedicine service with multiple modules, including 12-lead ECG signal processing, ECG report visualization with management, and ECG e-learning, is created in the datacenter of Microsoft Windows Azure platform. The Window Azure provides the environment of .NET programming development platform, data storage, Microsoft SQL-like database management, and the use of virtualized hardware resources. The first reason for adopting Azure platform is to avoid the situation of “data-lock-in.” In the event that hospitals do not use the cloud service, ECG data stored in cloud can be easily re-delivered to on-premise hospitals with MS-SQL databases, whose data structures are similar with Azure’s. The second reason for adopting Azure platform is because our developed programs of ECG processing, management, and visualization are based on our previously used .NET programming environment [6,7,18]. The 12-lead ECG processing module in the cloud can receive, process, and store ECG waveform data generated by the text based XML-ECG, the binary based SCP-ECG, and DICOM-ECG, which are frequently used ECG data formats and usually used in urban hospitals, rural clinics, or ambulances via the internet. The 12-lead ECG visualization and management modulus provide clinicians with ubiquitous 12-lead ECG report queries and diagnoses by the web browser via internet with the uses of computers or cell phones. In addition, these cloud based 12-lead ECG processing, visualization, and management can function as utility computing to assist rural clinics without PACS, HIS, vendor-specific ECG viewers or additional telemedicine equipments. In this study, two military hospitals in Taiwan form a 12-lead ECG network where these two hospitals can interoperate 12-lead ECG records, real-time tele-consultation between the two hospitals, pre-hospital ECG diagnosis, ubiquitously diagnose 12-lead ECG and continuing ECG e-learning via our cloud services.

### The cloud Azure

**Figure 3:**
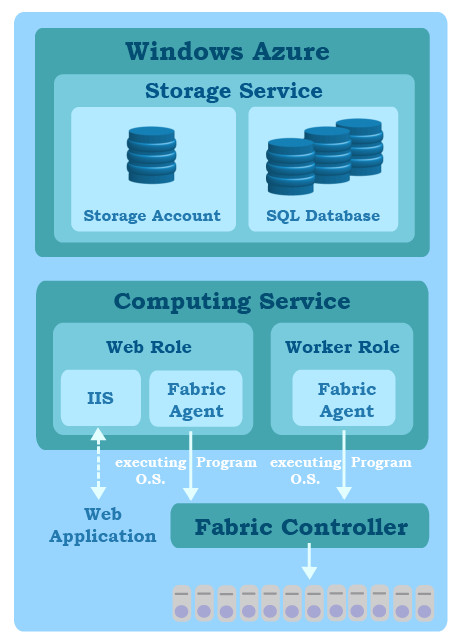
** The Windows Azure platform.** The user can implement applications in Azure’s Web roles and Work roles, and manage data in storage account or database. Azure’s Fabric controller can guarantee that the conducting applications are high availability.

Figure 3 illustrates the basic scheme of Windows Azure platform, which allows users to develop applications using .NET library, i.e., platform as a service (PaaS), and to create the software as a service (SaaS) in the cloud [27,28]. The running program on the Azure is represented by multiple Web roles and Work roles that are user-developed codes executing in the virtual machines (VM) of the Azure platform. The Web role serves to communicate with web-based applications located outside of the Azure through Internet Information Server (IIS) with Hyper Text Transfer Protocol (HTTP). The Work role functions as a program running on the background of the Azure platform. The communications between Web roles and Work roles are dependent on the queue, which is a special data structure provided by the Azure and is used as a message passing among various roles. Each role on the Azure is installed with a Fabric agent, which reports the working status of computing resources and the remaining capacity to a Fabric controller. The computing resources and the remaining capacity of a running program on the Azure are monitored by the Fabric controller which manipulates the instances of roles and physical resources in the datacenter in response to a job loading. As also shown in Figure 3, the Azure provides users with two kinds of data storage services. The first one is a data storage place where data are accessed by a virtual universal resource locator (URL) link with the secret key protection and the user’s authentication. The second one is Structured Query Language (SQL) based Azure database that allows users to manipulate relational entities by using traditional SQL.

### 12-lead ECG telemedicine service with enhanced security and privacy protection

**Figure 4:**
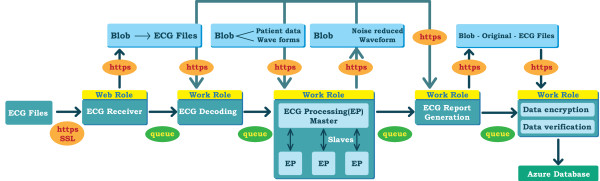
** The cloud 12-lead ECG telemedicine service with enhanced security.** This service is performed through four steps, including ECG files receiving, ECG file decoding, ECG waveform processing, and ECG report generation. It should be noted that the data passing among roles and data storage are encrypted and verified by a protected key. In addition, the use of web roles and work roles is authenticated with authorized users.

Figure 4 illustrates the 12-lead ECG telemedicine Service design in Windows Azure. This ECG telemedicine service includes the following four stages. First, an ECG receiver represented by a Web role via internet is used to receive the incoming ECG files generated from clinically-used ECG instruments, such as XML-ECG, SCP-ECG, and DICOM-ECG, in hospitals or in ambulances. Second, an ECG decoding program represented by a Work role is used to extract the 12-lead ECG waveform data and the specific patient data from received ECG files. Third, a parallel ECG signal processing program running with the master-slave mode represented by three Work roles is used to reduce the extracted 12-lead ECG noises and artifacts. Fourth, an ECG report generator, and a data encryption with data verification program represented by two Work roles are used to safeguard patients’ ECG reports in the Azure database. In this study, the authorized users can ubiquitously perform 12-lead ECG tele-diagnosis via Web access. Also, an ECG E-learning database is created to collect clinical 12-lead ECG reports which may be used as e-learning materials to enhance clinicians’ diagnostic skills. As the same design schema of Figure 4, a Web role is used to connect clinicians’ queries of ECG learning cases and database via web access.

To safeguard the ECG data in the cloud, we have designed a security mechanism to prevent them from theft and protect the patients’ privacy, including authentication for the use of Web roles and Worker roles, data encryption during message communications among roles, and ECG file encryption and verification while ECG report are retrieved in storage account and database. As also shown in Figure 4, ECG files are transmitted via secure sockets layer (SSL) based HTTP (HTTPS) where ECG files are protected by certificate based encryption and verification instead of plain text based HTTP. During this first stage, these received ECG files are stored temporarily in a secret key protected storage account as a big object (Blob), which is a special data format in the Azure. In the same time, to reduce the possible malicious attacks from people with currently-used Azure accounts, the ECG receiver represented by a Web role is limited to the exclusive use of recognized hospital internet protocol addresses (IPs). During the second stage, as ECG files are received successfully, the ECG decoding program is activated by the previous Web Role-ECG receiver through the notice of the queue, which is also a special data format in the Azure. The ECG decoding program retrieves the received ECG files stored in a storage account via the virtual web address link with HTTPS to extract 12-lead ECG waveform data and the other relevant patients’ data. During the third stage, the ECG signal processing Work roles are activated by the previous ECG decoding Work to retrieve the extracted ECG waveform data stored in the storage account to conduct ECG noise reduction. To gain speed of ECG processing, the extracted 12-lead ECG waveform data are divided into several parts and then spawned to the Work roles activated simultaneously with the master-slave mode to perform ECG noise reduction. The master Work role in ECG noise processing collects the noise-removed ECG waveform data from the slave Work roles and generates a clinical styled ECG report and an original ECG file with noise-reduced waveform data. During the fourth stage, a data encryption program is activated to encrypt the generated ECG files before the ECG files are stored in the database, and a data verification program is also activated to generate message digests of ECG files to ensure the integrity of ECG files placed in the database. The encrypted ECG files with generated message digest are then stored in the Azure database to ensure data integrity and confidentiality in the cloud. Thus, the patients’ identified code and disease records shown on the ECG report is protected.

### 12-lead ECG visualization and management

**Figure 5:**
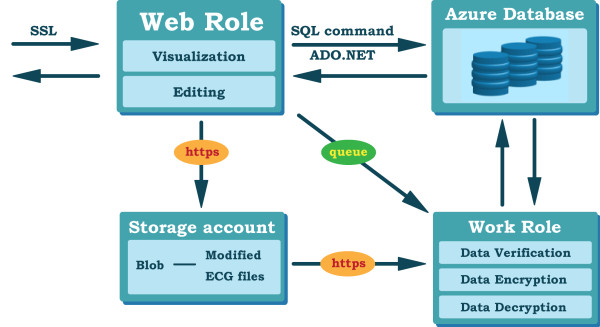
** The implementation of ECG visualization and management functions in cloud Azure.** A Web role and a work role are created to provide clinicians with the services of 12-lead ECG report queries and editing via Web access. The queried ECG reports stored in Azure SQL database are decrypted and examined by a data verification program. This ECG file can be visualized by vector graph visualization, and allows clinicians to edit ECG files with simultaneous updating on the original ECG file.

As shown in Figure 5, a Web role is created to provide clinicians with the services of 12-lead ECG report queries and editing via Web access. The Web role receives clinicians’ ECG queries over HTTPS and redirects queries to Azure SQL database with secured Tabular data stream (TDS) via ADO.NET. The queried ECG reports are decrypted and examined by a data verification program represented by a Work role to determine whether the ECG files are intact without malicious modifications in the Azure database. This ECG file visualized by Silverlight, a .NET library for vector graph visualization, allows clinicians to edit ECG files with simultaneous updating on the original ECG file. Before being saved in the Azure database, the modified ECG files are encrypted with message digests through Work roles implemented with encryption and verification programs as the stage 4 of the 12-lead ECG processing service.

An ECG E-learning database is created to collect clinical 12-lead ECG reports which may be used as e-learning materials to enhance clinicians’ diagnostic skills. As the same design schema of ECG visualization and management, a Web role is used to connect clinicians’ queries of ECG learning cases and database via web access.

## Results

### Practice and evaluation of ECG tele-consultation

Based on the views of senior cardiologists in Taoyuan Armed Forces General Hospital in Taiwan, a detailed 12-lead ECG report, including the description of the patient’s present symptoms, his/her medical history, and medication records, is crucial for a cardiologist who provides emergency ECG tele-consultation for the consulting clinicians. Among all the relevant medical information, the past and current ECG records are the most important references for clinicians to determine whether the patient is at an immediate risk of dying [29,30]. In almost all the hospitals in Taiwan and in many other countries, the medical staff cannot make additional notes or edit 12-lead ECG files generated from ECG devices. In addition, most rural clinics are unable to purchase expensive vendor-specific ECG software to store electronic ECG records, nor can they purchase HIS to manage patients’ medication records. To facilitate long-distance ECG tele-consultation, this cloud based 12-lead ECG telemedicine service can allow clinicians to store, browse, and interactively edit 12-lead ECG reports via web access while tele-consultation is taking place.

**Figure 6:**
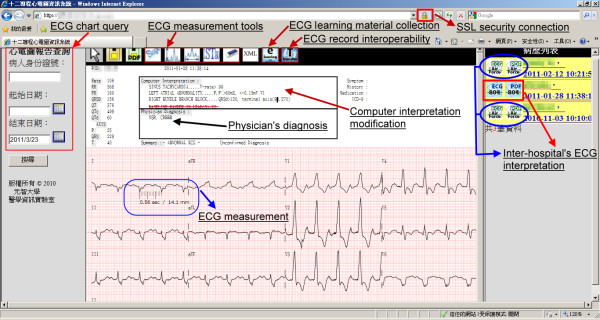
** The web accessed 12-lead ECG visualization and management service in the cloud.** The physicians can easily diagnose the past and the present ECG charts of the patients transferred from other hospitals via ECG chart exchange in the cloud.

Figure 6 shows the use of 12-lead ECG telemedicine service of an on-site physician on a Windows based desktop computer via web access, including ECG report queries, ECG waveform measurement, ECG interpretation, record management, patients’ symptoms entry, disease history description, and medication record editing , This edited report will be stored in a centralized cloud database. If a patient is transferred to the other hospital or emergency tele-consultation that is on demand, the remote cardiologists can easily and quickly browse all of the patient’s ECG records, and medication records via this collaborative cloud service.

This 12-lead ECG cloud service enables hospitals to store and manage patients’ ECG records via web access through the internet connection of clinically-used 12-lead ECG instruments. In this way, urban hospitals and rural clinics can easily use the common service for effective inter-hospital ECG tele-consultation without any costly installation of hardware or software on-premise.

### Practice and evaluation of ECG tele-diagnosis via cell phone

Using online services via cell phones to deliver or retrieve ECG reports can effectively realize ECG telemedicine. Even when a senior cardiologist is not at the hospital, this cardiologist can timely respond to the consultation request to offer ECG interpretation and decision making support via cell phones and this cloud ECG service.

**Figure 7:**
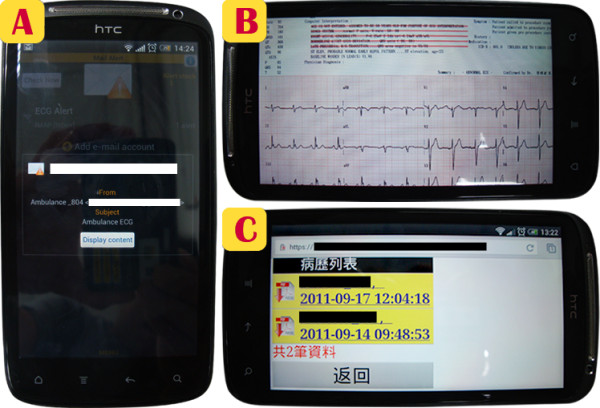
** 12-lead ECG tele-diagnosis via cell phone.** Using 3G wireless telecommunication via cell phones to deliver or retrieve ECG reports can effectively realize ECG tele-diagnosis.

As shown in Figure 7, an Android based mobile phone, HTC sensation with a CPU speed of 1.2 GHz and the memory capacity of 768 MB, is used to receive ECG reports from cloud Azure. The panel A of Figure 7 shows the cell phone with email alert notifications delivered from cloud ECG services to remind the off-site expert cardiologist to respond to an emergency email where patients’ ECG reports are attached. As illustrated on the panel B of Figure 7, the off-site expert cardiologist can easily download the attached 12-lead ECG reports, interpret the ECG, and instruct on-site physicians to prepare for the appropriate treatment via his cell phone. As shown on the panel C of Figure 7, the off-site cardiologist can use his cell phone to browse past and present ECG records of the same patient via cloud ECG database connection.

To evaluate the time needed for a clinician to receive ECG files via his mobile phone from the cloud, 100 trials of data transmission from the cloud to the mobile phone are conducted. To measure the average time to brows an ECG report on a mobile phone, a “trigger” is pre-implemented in a mobile phone. This “trigger” is a procedural SQL code which is automatically invoked by the mobile database, upon the occurrence of a data manipulation event. For example, when a cardiologist issued an ECG report query command, the trigger starts the timing until the requested ECG is successfully delivered to the mobile phone. The average time for browsing an ECG report without data verification in the cloud is approximately 5.65 ± 0.38 secs, but the average time for browsing the file with data verification in the cloud is approximately 6.28 ± 0.13 secs. While these telemedicine applications ensure data security, the mechanisms of data encryption, decryption and verification in the cloud and in the process of being delivered to the mobile devices slightly delay data visualization and thus have a little toll on the time efficiency of the programs.

### The cloud based 12-lead ECG e-learning

12-lead ECG continuing education is important in clinical practice, especially for clinicians who need to improve ECG interpretation skills. Traditional ECG continuing education courses are provided by experienced cardiologists in urban hospitals; consequently, rural physicians lack opportunities to receive training. This 12-lead ECG telemedicine service enables clinicians across hospitals around the world to collect, to review, and to discuss large-scale ECG reports for the education and training purposes. Clinicians can even perform independent learning ubiquitously from the cloud database.

**Figure 8:**
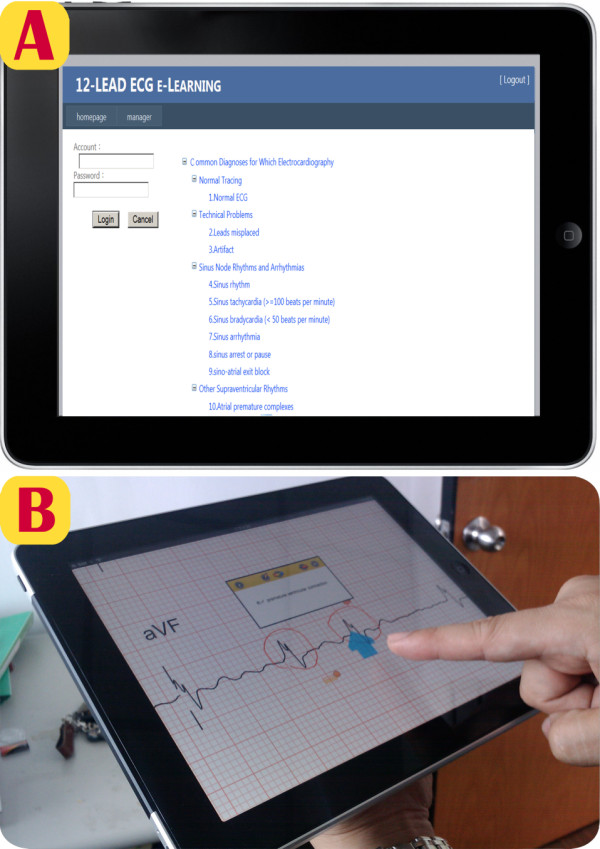
** A cloud computing based 12-lead ECG e-learning service.** Clinicians can perform independent ECG learning ubiquitously from the cloud database.

As shown on the panel A of Figure 8, clinicians can download ECG files, which have been categorized based on the recommendation of American College of Physicians, via IPad for mobile learning [31]. As also shown on the panel B of Figure 8, clinicians can denote the reports and annotate the finding via IPad for self-learning.

In addition, this cloud telemedicine service can become a valuable asset for advanced research in tele-cardiology, because clinicians from different hospitals can upload or download clinical ECG files with interpretation and request additional consultation.

## Discussions

### 12-lead ECG interoperability

This study implemented a cloud computing based telemedicine application, which can upload 12-lead ECG reading to a cloud service from which they can be visualized on a variety of network connected devices, both mobile and fixed. This cloud application supports a variety of clinically used 12-lead ECG devices, such as Philips XML-ECG, HP compatible SCP-ECG, and Mortara DICOM-ECG by first transcoding the data from a vendor-specific format to an XML format in a public cloud computing platform, Azure.

### Data security in the cloud

To safeguard data stored in cloud, most cloud providers offer security mechanisms including filtering unauthorized users, auditing abnormal data retrieval actions, and preventing data from theft by hiding actual locations of data in the cloud [32]. In addition, users’ cloud data are automatically replicated and distributed in different locations in the datacenter and can thus be retrieved even if a natural or man-made disaster occurs. However, “lack of trust” remains the major issue that keeps consumers from placing sensitive data in the cloud [33].

### Data backup in hospitals and cloud

In this study, the cloud Azure functions as a convenient and automatic database backup service, which constantly replicates data from the cloud and resends the replicated data to the on-premise. In this way, users can always retrieve their data even in the event of internet breakdown. The data stored in the Azure database can be synchronically backed up to MS-SQL database on-premise via commonly used ADO.NET technology, which allows data query and data modification between SQL database.

### Enhanced ECG data security and privacy protection

To avoid malicious internal data theft in the cloud datacenter, we designed an enhanced security mechanism in the Azure cloud to protect patients’ privacy as shown in Figure 4, and Figure 5. The ECG data in the processing and the storage stages are well protected by users’ authentication, encryption, and verification. In this way, the cloud provider cannot access the processed ECG data and the ECG reports without the keys owned by hospitals.

To protect patients’ privacy in the cloud, each file is represented by the patient identification code made of a software generating string, which is known only by the hospital. This patient identification code differs from the commonly used personal identification number or the health insurance number.

Currently used mobile telecommunication services do not guarantee data security or patients’ privacy, because not every access point in the mobile network can be trusted. To enhance the security of ECG data transmitted via cell phones, the mobile ECG data transmission is performed through a specific network provided by the mobile communication service provider, and this network is used only for data transmission between the off-site physician and the on-site clinicians. In our study, the off-site physician’s cell phone is authenticated as the authorized user in the hospital, who can obtain an IP to gain access to the cloud service. Meanwhile, the delivery and retrieval of ECG data are protected by SSL and the exclusive network service.

### The cost-effective ECG telemedicine service

Before the hospital adopts this cloud 12-lead ECG telemedicine service as recommended by cardiologists, the cost of this cardiac care service should be minimized and the efficiency of data management needs to be maximized at the same time. Traditionally, client/server based computing requires costly installation of software and hardware on-premise. As a contrast, this newly developed cloud computing has the following two advantages: (1) the massive computing resources can be used as the model of pay-as-you-go without long-term commitment, and (2) the cost of data storage and data management is low. Based on Azure’s pricing model, the cost of this cloud service is based on (1) the use of CPU resources, which is charged by USD $0.12 per hour, and (2) the use of database, which is charged by USD $9.99 per GB per month.

In general, not only do rural clinics have difficulty affording expensive computerized 12-lead ECG software and ECG telemedicine instruments, it also may not have ITs to maintain computerized data. Meanwhile, urban hospitals do not want to purchase extra ECG telemedicine station to receive specific ECG measured in an ambulance. With the aforementioned two advantages of cloud computing, the realization of 12-lead ECG tele-consultation between rural clinics and urban hospitals and the performance of pre-hospital ECG diagnosis from the ambulance can take place easily and inexpensively. With the use of this 12-lead ECG telemedicine cloud service, tele-consultation and pre-hospital ECG diagnosis can be performed on demand at anytime from anywhere. Importantly, this service is inexpensively charged on the basis of pay-per-use.

### The difficulty of the 12-lead ECG telemedicine

Despite the progress of mobile computing and the benefit of applying m-health onto cloud computing, the development of 12-lead ECG telemedicine is impeded by heterogeneous vendor-specific ECG data formats where ECG waveform encoding rules are unknown. If the open ECG standards with open ECG waveform encoding rules, such as SCP-ECG, DICOM-ECG, and XML-ECG, can be adopted by most ECG manufactures, ECG interoperability and telemedicine applications can be better realized.

The advantage of SCP-ECG is the size of 15 KB ~ 30 KB with compressed ECG waveforms. Therefore, SCP-ECG can be used to deliver ECG without being limited by the bandwidth of mobile phone network, and it can also save the storage charges in the cloud database. Moreover, SCP-ECG is capable of storing serial ECGs, which is useful for clinical ECG diagnosis. The major disadvantage of SCP-ECG is its binary structure, which cannot be easily integrated with text based PHR. On the other hand, DICOM-ECG with the file size of 250 KB and with uncompressed waveforms can be integrated with numerous medical images stored in PACS at the hospitals to enhance the efficiency of medical data management. However, the cloud application on data retrieval from PACS has not been fully realized because most hospitals usually do not allow remote clinicians to retrieve data stored in PACS, and the huge image database in PACS is expensive for cloud applications. As compared with SCP-ECG, XML-ECG with the size of 500 KB and with uncompressed waveforms might not be suitable for ECG delivery via mobile telephone network and for ECG storage in the cloud database. Nevertheless, text based XML format is human readable and can be easily integrated with patients’ PHR. Additionally, as compared with the binary based ECG formats, the cross-platform XML ECG document can be visualized and easily edited via web browsers and cloud application without the limitations of computer O.S. In conclusion, each of the above three open-standard ECG formats has pros and cons when it is used in ECG telemedicine. It is clear that open-standard ECG formats are superior to vender-specific ECG formats. To advance the clinical research of tele-cardiology, we need open ECG data formats, such as binary based SCP-ECG, DICOM-ECG, and text based XML-ECG. In this way, ECG interoperability can be better realized through the conversions among these open data formats.

## Conclusions

As compared with the traditional ECG tele-consultation, this cloud-based pervasive telemedicine service can realize interoperability across various mobile and fixed devices. It greatly enhances the convenience of ECG interpretation and the efficacy of tele-consultation, as it enables cardiologists to interpret ECG ubiquitously, to access patients’ current and past ECG records across hospitals via centralized cloud database, and to provide pre-hospital diagnosis in time. Apparently, this service advances clinical work and research on 12-lead ECG telemedicine with ECG interoperability, as it establishes an open tele-consultation platform from clinic to person and from hospital to hospital.

In this study, we create a new 12-lead ECG telemedicine cloud service enabling ubiquitous delivery of inter-hospital 12-lead ECG reports via cell phones. This developed 12-lead ECG telemedicine cloud service can improve 12-lead ECG telemedicine service quality. This service is mobile, convenient, easy to use, and effective, as it provides ECG tele-consultation via a common platform or offers timely pre-hospital ECG diagnosis based on the ECG records measured and sent from the ambulance. Importantly, this service smartly meets the demand of 12-lead ECG research and tele-consultation in clinical practice. This cloud computing based 12-lead ECG telemedicine system upgrades our previously developed applications, facilitates the collaboration of inter-hospitals, and enhances the efficacy of tele-consultation.

## Abbreviations

### Medical Informatics

ECG, Electrocardiography; SCP-ECG, Standard Communication Protocol–ECG; XML-ECG, Extensible Markup Language based ECG; DICOM-ECG, Digital Imaging and Communications in Medicine based ECG; HIS, Hospital Information System; PACS, Picture Archiving and Communication System; ISO, International Organization for Standardization; FDA, Food and Drug Administration; NEMA, National Electrical Manufactures Association; PHR, Personal Health Record; IIS, Internet Information Server; GSM, Global System for Mobile Communication; SSL, Secure Sockets Layer; HTTPS, Secured Hyper Text Transfer Protocol; SQL, Structured Query Language; TDS, Tabular data stream; URL, Universal Resource Locator; VM, Virtual Machine; PaaS, Platform as a service; SaaS, Software as a service; ED, Emergency Department; EMT, Emergency Medical Technician; EuMHA, European M-Health Alliance; PEM, Personal ECG Monitor; EPI-MEDICS, Enhanced Personal, Intelligent, and Mobile system for Early Detection and Interpretation of Cardiac Syndromes.

### Information Technology

ECG, Electrocardiography; SCP-ECG, Standard Communication Protocol–ECG; XML-ECG, Extensible Markup Language based ECG; DICOM-ECG, Digital Imaging and Communications in Medicine based ECG; HIS, Hospital Information System; PACS, Picture Archiving and Communication System; ISO, International Organization for Standardization; FDA, Food and Drug Administration; NEMA, National Electrical Manufactures Association; PHR, Personal Health Record; IIS, Internet Information Server; GSM, Global System for Mobile Communication; SSL, Secure Sockets Layer; HTTPS, Secured Hyper Text Transfer Protocol; SQL, Structured Query Language; TDS, Tabular data stream; URL, Universal Resource Locator; VM, Virtual Machine; PaaS, Platform as a service; SaaS, Software as a service; ED, Emergency Department; EMT, Emergency Medical Technician; EuMHA, European M-Health Alliance; PEM, Personal ECG Monitor; EPI-MEDICS, Enhanced Personal, Intelligent, and Mobile system for Early Detection and Interpretation of Cardiac Syndromes.

### Cloud Computing

ECG, Electrocardiography; SCP-ECG, Standard Communication Protocol–ECG; XML-ECG, Extensible Markup Language based ECG; DICOM-ECG, Digital Imaging and Communications in Medicine based ECG; HIS, Hospital Information System; PACS, Picture Archiving and Communication System; ISO, International Organization for Standardization; FDA, Food and Drug Administration; NEMA, National Electrical Manufactures Association; PHR, Personal Health Record; IIS, Internet Information Server; GSM, Global System for Mobile Communication; SSL, Secure Sockets Layer; HTTPS, Secured Hyper Text Transfer Protocol; SQL, Structured Query Language; TDS, Tabular data stream; URL, Universal Resource Locator; VM, Virtual Machine; PaaS, Platform as a service; SaaS, Software as a service; ED, Emergency Department; EMT, Emergency Medical Technician; EuMHA, European M-Health Alliance; PEM, Personal ECG Monitor; EPI-MEDICS, Enhanced Personal, Intelligent, and Mobile system for Early Detection and Interpretation of Cardiac Syndromes.

### Others

ECG, Electrocardiography; SCP-ECG, Standard Communication Protocol–ECG; XML-ECG, Extensible Markup Language based ECG; DICOM-ECG, Digital Imaging and Communications in Medicine based ECG; HIS, Hospital Information System; PACS, Picture Archiving and Communication System; ISO, International Organization for Standardization; FDA, Food and Drug Administration; NEMA, National Electrical Manufactures Association; PHR, Personal Health Record; IIS, Internet Information Server; GSM, Global System for Mobile Communication; SSL, Secure Sockets Layer; HTTPS, Secured Hyper Text Transfer Protocol; SQL, Structured Query Language; TDS, Tabular data stream; URL, Universal Resource Locator; VM, Virtual Machine; PaaS, Platform as a service; SaaS, Software as a service; ED, Emergency Department; EMT, Emergency Medical Technician; EuMHA, European M-Health Alliance; PEM, Personal ECG Monitor; EPI-MEDICS, Enhanced Personal, Intelligent, and Mobile system for Early Detection and Interpretation of Cardiac Syndromes.

## Competing Interests

The authors hereby declare that there were no competing interests.

## Author Contributions

Jui-chien Hsieh, Ph.D. was involved in plan and design of this study. Meng-Wei Hsu was involved in this cloud programming. All authors read and approved the final manuscript.

## Authors’ information

Dr. Jui-chien Hsieh, the director of Medical Informatics & Telemedicine Lab, works as an assistant professor of the Information Management Department at Yuan Ze University in Taiwan. He obtained his Ph.D. and M.S. degrees from the program of Biomedical Engineering at Rutgers University, New Brunswick, New Jersey, U.S.A.His research interests include medical informatics, computational biology, and pervasive health. Dr. Hsieh’s contact address is the Department of Information Management, Yuan Ze University,135 Yuan Tung Road, Chungli, Taoyuan 320, Taiwan. Dr. Hsieh’s email address is http://jchsieh@saturn.yzu.edu.tw.

## Pre-publication history

The pre-publication history for this paper can be accessed here:

http://www.biomedcentral.com/1472-6947/12/77/prepub
